# Promoting Wellness in Orthopaedic Surgery Residency

**DOI:** 10.5435/JAAOSGlobal-D-21-00227

**Published:** 2022-03-07

**Authors:** Vincent Federico, John Higgins, Michael Nolte, Monica Kogan

**Affiliations:** From the Rush University Medical Center, Chicago, IL.

## Abstract

The challenges associated with surgical residency have been well documented and described. Despite implementation of work-hour restrictions, residency remains a balancing act between patient care, surgical and clinical education, medical record documentation, and research endeavors. The added challenge of balancing these duties with life outside of the hospital further complicates the situation. Multiple studies have documented the stress associated with residency training, highlighting the prevalence of emotional exhaustion, detachment from people both in and out of the hospital, and a concerning rate of clinical depression among residents. Moreover, this emotional exhaustion has been shown to directly correlate with residents' clinical performance and abilities to carry out academic duties. More recently, feelings of isolation and detachment have been exacerbated by the necessity of COVID-19 precautions and change in clinical duties. The Accreditation for Graduate Medical Education (ACGM) now requires all residencies to include programming that focuses on resident well-being. Programs have implemented various strategies in an effort to help their trainees cope with the stress associated with residency and beyond. Despite the variety in approaches of programs, these initiatives have the similar objective of teaching resilience and the ability to navigate stressors in a healthy and effective manner. The programming can also serve to promote resident interaction and develop camaraderie in an effort to minimize feelings of emotional exhaustion and isolation. In this article, we discuss the importance of sustained physician wellness and describe approaches from various professions that can be implemented into the wellness curriculum for residency programs across the country. By promoting a culture of wellness and incorporating effective wellness programming, our aim is that residents will be able to succeed not only in their training but also in their personal lives and professional endeavors after graduation.

The challenges of a surgical residency are well documented.^1^ Balancing electronic medical record documentation requirements, patient care, research endeavors, and balancing life outside of residency can be a source of notable stress on the learner.^2^ The pernicious effects of these postgraduate years in training have also been reported, mostly in the form of studies on physician burnout rates. Burnout is a syndrome characterized by depersonalization, exhaustion, and a reduced sense of personal accomplishment^.3^ It was thought that the duty hour restrictions would lead to an improvement in the incidence of burnout and fatigue; however, even after the implementation of these restrictions, burnout and fatigue are still prevalent among residents.^2,4^ About orthopaedic surgery in particular, the rate of burnout among residents has been cited anywhere between 38% and 56%.^5-7^

This high potential for emotional exhaustion and lack of fulfillment has contributed to diminished clinical and academic performance. For example, a recent study showed that performance on the Orthopaedic In-Training Examination was worse among residents exhibiting burnout symptoms, even when controlling for test-taking ability.^8^ Moreover, physician burnout has been associated with a higher likelihood of medical errors.^9,10^ Despite the increased autonomy, decreased call hours, and further proficiency in surgical skills with being an attending, the symptoms of fatigue and detachment from work may still be occur. Several studies demonstrate the high stress levels associated with being an orthopaedic attending, further emphasizing that the causes of burnout continue after training.^3,11-13^

A reasonable question arises: “How do we help the ones who are supposed to help everyone else?” Physician wellness can be a difficult topic to address because of the subjectivity of what each individual considers to be beneficial to their well-being and the constraints of a busy schedule. The importance of this concept in resident education was emphasized in 2017 when the Accreditation for Graduate Medical Education (ACGM) required wellness programming for all residencies regardless of specialty. Furthermore, the impact of the COVID-19 pandemic on resident wellness has been profound and further emphasized the need to address mental health and well-being from early training to practice as an attending.^14^ In this article, we highlight successful initiatives that have been undertaken by other orthopaedic surgery programs, programs in other medical specialties, and professions outside of medicine and offer suggestions that can hopefully act as first steps in improving resident wellness around the country (Table 1). By increasing the awareness of different resident wellness initiatives, programs can develop their own approaches that will allow their learners to develop the skill sets to succeed in residency and have a foundation to approach stressors in practice, ultimately diminishing the likelihood of burnout and depression.

**Table 1:** Wellness Goals and Examples of Common Strategies

Increasing mindfulness	• Hold quarterly meetings as a residency to discuss common stressors and brainstorm opportunities for improvement.^36^ • Set aside time for nonclinical team-building activities such as service outreach and communication-based competitions.
Promoting “grit”	• Celebrate examples of resident perseverance through challenging experiences through public recognition and hospital award nomination. • Identify residents who may be struggling to thrive in the face of adversity, providing support and encouragement.
Leading by example	• Empower senior residents with a positive track record to lead the junior classes • Hold regular resident-only meetings to discuss current concerns and issues from which specific issues can be vocalized to leaders who can implement changes^28^ • Consider devoting a specific position to oversee the wellness in a program with initiatives such as mentorship, in-person events, and professional development classes^15,30,31^
Communication	• Encourage face-to-face dialogue and discussion among residents and faculty, as permitted. • Reduce the amount of time focused on a screen where distractions can easily arise.
Workplace diversity	• Aim to attract diverse incoming residents from various backgrounds, programs, and life experiences to broaden the horizons and perspectives of the residency as a whole. • Develop a resident sibling program in which residents are asked to stay in routine contact to build stronger longitudinal relationships.
Physical wellness	• Give residents the ability to exercise and engage in outside activities. • Support access healthy dietary options.^33,35,49^

## Wellness in Orthopaedic Surgery Residency

Despite the physical, mental, and emotional toll that orthopaedic surgery training places on residents, the importance of resident well-being cannot be overlooked. To effectively treat patients, physicians must be both physically and mentally healthy. As discussed by Carter et al,^3^ there are two keyways to promote physician well-being—either to focus on improvement in the physician themselves or to encourage change in the systems in which the physician operates. This was further substantiated in the systematic review and meta-analysis completed by West et al,^15^ in which both studies recognized that individual-focused and organizational interventions can have positive effects on physician wellness, the most effective of which were mindfulness or focused stress management. Although the combination of these two strategies has not been explicitly studied, it can be presumed that addressing both the physician and the medical system can produce a synergistic effect. However, many of the sources of burnout and frustration stem from government or institutional requirements (e.g., electronic medical record and hospital protocol requirements), and changing the system may be extremely difficult. As so, focusing on the factors that can be controlled and teaching resilience can be a first step in improving physician well-being.

In an ideal world, the attributes that make physicians successful and resilient would have already begun to be developed before starting residency. Grit, a positive noncognitive trait that is based on an individual's passion for a particular long-term goal, coupled with a powerful motivation to achieve his or her objective, has been shown to be a greater predictor of success than intelligence.^16^ When combatting the many instances of failure or setback in residency, the ability to promote resilience among learners and colleagues is one of the most beneficial ways to improve confidence and prevent feelings of inadequacy. Higher grit scores have correlated with greater well-being of surgeons, a lower surgical resident attrition rate, decreased burnout for attending surgeons, and higher rates of career satisfaction.^16-18^ Even before starting residency, orthopaedic applicants have been shown to score in the 70th percentile for grit assessment when compared with the general population.^19^ However, grit is not necessarily a trait that spans all areas of a person's life because an individual may display grit in certain instances over others. For example, a collegiate athlete who required grit, perseverance, and determination to succeed in his/her/their athletic career may fall short in performance during residency. This can also be seen in residents who succeeded in medical school but start residency and the drive and grit that was seen as a student is gone. The hope, however, is that by nurturing and further developing the grit that allowed residents to succeed in medical school and fostering a sense of belonging, connection, and purpose, the learners will develop their own coping skills necessary to succeed in the inevitable difficult situations that will arise within their careers.

Although the ACGME now requires residencies to have programming that promotes resident wellness, it can be challenging to accomplish effectively because the definition of wellness can vary widely among different parties. Webinars and online presentations are in abundance. However, if there exists a lack of feeling connected to coworkers and patients, a lack of sense of purpose, the online programs may not address hardships faced by individual residents within a unique specialty, and these resources may not be as effective as intended. Although the information presented may be helpful in some ways, the online resources may not be able to address the larger issues that often times need more than a web-based presentation.

Simon Sinek in “Leaders Eat Last” describes that if people are left in an environment where they have to fend for themselves, even a “good day” at work may not be so good. He further discusses that sharing hardships brings people closer and results in a release of oxytocin in the brain.^20^ In essence, sharing hardships makes us feel better. It is ironic that in a world where the ability to be connected to one another is constant, people still feel disconnected and alone. In residency, this feeling can be exponential, given the long hours and the inability to see those outside of work on a regular basis. Essentially, as described by Krall^21^ in his article on physician wellness, the busier one is, the more one needs collegiality.

The feeling of isolation can be made worse by the constant reminder (e.g., social media) of what others are doing outside the hospital. This concept has been well documented in high school and college students.^22^ Promoting an environment where residents need to and are encouraged to communicate with each other can be crucial to fostering connection. Simple tasks such as a “no phone conference,” where residents put their phones away while in the conference room, serve to encourage residents to talk to one another versus spending time on their mobile devices. Furthermore, this concept does not need to end in residency. It can be brought to the professional setting at departmental meetings or board rooms so that colleagues can converse with one another, ask about their families, discuss cases or troubles, or enjoy being in the presence of others. The feeling of being connected is of the utmost importance, and the simple task of putting the devices away can help in fostering a sense of belonging. Feelings of being isolated can potentiate the feelings of fatigue and unhappiness. In a world where we have every avenue to be connected, the devices are not enough, people hunger for the human connection.

The lack of control of external surroundings has also been found to be an issue for residents.^23^ Put simply, less control leads to more stress. Although there are aspects of training that residents are unable to control, understanding that there are ways to have their voices heard may help with this feeling of despair. Resident-only meetings where discussions are held in a judgement-free environment can be held every few months. In these meetings, issues, concerns, or ideas for improvement within the residency can be discussed. The resident presenting is given a “safe space” for the discussion, and coresidents have the opportunity to learn from others' experiences and perspectives. These meetings can also foster a bond among residents who can provide an outlet when feelings of burnout begin. Quarterly small group resident meetings with the Program Director or Chairperson can give residents another opportunity for their voices to be heard, as well as a chance to have exposure with program leadership in a casual nonjudgmental setting.

Wellness and burnout prevention can also be further promoted by maintaining a focus on the groups most likely to be affected from the symptoms. In the 2011 survey by the American Orthopaedic Association, 384 orthopaedic residents reported a 56% burnout rate, with the highest risk being seen in those in their postgraduate (PGY) 2-year, female residents, and larger programs (defined as having six or more residents per year).^7^ By focusing on each of the surrounding factors predisposing these groups to higher rates of work dissatisfaction and emotional withdrawal, the hope is to encourage well-being early and effectively. During the PGY-2 year, residents at most programs experience a notable increase in independence through on-call duties and an emphasis on developing orthopaedic knowledge and surgical skills. As such, more frequent check-ins with faculty and chief residents, as well as initiatives such as group activities, volunteering, and access to both mental and physical health resources are all ways to help in this transition.

In addition, underrepresented minorities (URM) in orthopaedic surgery face other challenges during this transition that can potentiate the sense of burnout. Resources to promote their wellness both in and out of work can be instrumental in developing a strong sense of identity, belonging, and mental well-being. Introductions to women or URM mentors in orthopaedic surgery or general surgery can serve as an important resource for these residents. Studies have shown that mentorship in the medical profession leads to more successful and impactful mentees.^24^ Having a mentor who has experienced similar challenges in their surgical training can be helpful for women and URM residents, but it is not a necessity. Sambunjak et al^25^ completed a systematic review analyzing the effect of mentorship in the medical setting and found that the mentor's sensitivity was the most impactful quality in a mentor-mentee relationship, as opposed to matching race, sex, or religion. The role of mentors in medicine, and especially in orthopaedic surgery, cannot be understated and are pivotal in developing successful, well-trained, and healthy residents, especially for those who are not in the majority.

For some programs, incorporating team bonding experiences may be challenging because of funding; however, volunteer activities at the hospital or local organizations can give residents the feeling of purpose while simultaneously building connections with fellow residents while not causing a financial burden on the program. Furthermore, scheduling these activities in the place of a resident conference avoids infringing on time outside the hospital or on activities that are another source of wellness for the residents. Some Graduate Medical Education (GME) programs are incorporating wellness days each year to be used so that in the event a resident is sick, he/she/they will not need take a vacation day. Simple tasks such as these may be beneficial to prevent feelings of isolation and feelings of being overwhelmed. Encouraging wellness events that promote diversity and inclusion may be a step to developing resilient, productive residents and the development of healthy traits that can be used throughout residency and beyond.

As important as addressing the physician on the individual level is, the need to foster a culture of inclusion and wellness within a healthcare system is crucial. Creating an environment that offers perpetual opportunities to recognize and treat burnout is paramount. As Bohman et al^26^ describes, creating a culture of wellness can result in physicians who feel supported by their organizations and have a more efficient practice. Thus, by focusing on physician themselves, the benefits of improved patient care, positive morale among the members of a practice, and a development of a system that will benefit all stakeholders can be emphasized. At OrthoCarolina, for example, a medical director position was created to focus specifically on developing and sustaining a culture of wellness. Moreover, this individual has been given the resources and support necessary to implement positive changes to the system. Some examples were a formalized mentorship program, class dinners, and leadership education leading to improvement in physician engagement, resilience, and ability to disconnect and recharge outside of work.^27^ These types of programs should be strongly considered by all orthopaedic residencies and practices to promote wellness. Forums that emphasize honesty and vulnerability, such as one-on-one meetings with attendings/program leadership and open conferences/seminars among the entire residency program, can begin to create a system that operates in support of the resident.

The Stockdale Paradox referenced by Jim Collins in “Good to Great” describes a philosophy of facing hardships head on with grit, faith in perseverance, and honest expectations to prevail even during the hardest of circumstances.^28^ As previously mentioned, junior residents face notable challenges in their early training. The philosophy of the Stockdale Paradox can help residents prevail through the utilization of grit, determination, and realistic, honest expectations of their training. Residency programs can help in this by working to develop of strong, successful, and psychologically healthy residents through effective wellness programming, giving the learners the skillset to prevail during residency and beyond.

## Wellness Initiatives in Other Medical Residencies

Since the implementation of the updated ACGME Common Program Requirements for all residencies and fellowships in 2017 to include measures to address resident psychological, emotional, and physical well-being, residency and fellowship program directors across medical specialties have been looking for the most effective ways emphasize resident well-being. There is a need to not only identify when physician trainees are experiencing a need for intervention but also implementing initiatives that are both individually tailored to each resident's needs and capable of providing institutional change that promotes acceptance of physician well-being. Krall,^21^ in his article, describes the 10 commandments to physician wellness. He includes the emphasis on self-reflection, self-care, and knowing one's limits. Self-awareness is an excellent place to start regarding teaching residents a practical guideline to follow in their careers.

More recently, Kashat et al^29^ described a method in otolaryngology residents by which they performed a needs assessment in the form of an anonymous survey to each of their residents asking them to rank wellness topics in order of importance. This was then followed by an open dialogue focus group that helped to determine systemic barriers to wellness. By involving the residents in the creation of the initiatives, it allows for the implementation of both immediate and long-term modifiable factors that are direct responses to resident stressors. This methodology of resident inclusion is an easily implemented way for all residencies to ensure they are focusing on what their individual residents require. Other studies have also demonstrated the positive impact that wellness initiatives can have on physician burnout rates even if not resident-driven. Specific examples include individual chief resident check-ins, facilitated discussion, mindfulness training, narrative or artistic reflection, and appointment of dedicated faculty or house staff members as wellness champions within each residency.^30^

In addition, the importance of resident physical health cannot be de-emphasized. In a survey of 4999 obstetrics and gynecology residents, exercise was the only common practice residents participated in that was associated with a notable reduction in report of any wellness problem (OR 0.68, *P* < 0.001).^31^ Moreover, the potentially harmful nutrition habits of physicians, including poorly balanced diets and eating habits, inadequate hydration, nutrient deficiency, and caffeine use may further be hindering optimal emotional, physical, and cognitive performance.^32^ These studies highlight the importance of resident access to on-site or nearby gyms, resident-sponsored group or individual physical activities, access to healthy food and drink options throughout the day, and encouragement by faculty and senior residents for using these resources during the workday.

## Wellness Initiatives in Other Professions

The American workforce experiences stress in similar ways that orthopaedic surgery residents do in the form of increased work hours, physically demanding tasks, and emotionally straining projects. In addition, these work-related stressors have a compounding effect on workers' lives outside of the office, leading to reduced physical activity, unhealthy dietary habits, and poor mental well-being. Expectantly, the culmination of these factors leads to decreased productivity while at work, increased absenteeism, and poor physical health. The American Psychological Association estimated the financial ramifications of work-related stress to be between $221.13 million and $187 billion.^33^ To combat these deleterious effects, companies have invested in stress-management departments and programs to provide various options to reduce employee stress. Corporations, small to large, recognize the need for comprehensive wellness programs to be more profitable and reduce potential losses.

Similar to medicine, the academic and corporate worlds have identified wellness initiatives as key opportunities for both individual and group improvement. Horton and Snyder proposed seven dimensions of wellness critical to success in both the academic and corporate realm: intellectual, emotional, social, environmental, occupational, physical, and spiritual.^34^ They subsequently quantified these dimensions for 249 college students and analyzed them in the setting of grade point average to determine which realms were associated with success. They found that top performing students based on grade point average spent markedly more time on physical and environmental activities in particular when compared with their peers. Physical wellness includes activities such as exercise, sleep, diet, and personal hygiene, whereas environmental wellness focuses on acknowledgement and improvement of one's surroundings, such as taking time to appreciate nature, cleaning, recycling, or gardening. Although it is difficult to quantify time spent in this environmental realm, it does seem to be a critical aspect of overall wellness.^35^

A *balance* of wellness dimensions is also critical. Many individuals in the academic and corporate realm have exhibited substandard performance despite high physical wellness scores, suggesting that it is possible to spend too much time in a single wellness dimension. Similarly, it was hypothesized that individuals who invested in the occupational dimension (nonacademic employment) would have low performance scores because of time spent away from academics. However, most low performers spent zero time on occupation wellness, further suggesting that a *balance* among the dimensions is key.

Adopting these measures in the medical realm can be challenging. Publicizing and making residents aware of these seven dimensions is the first step.^36^ Furthermore, having an awareness of how one is dividing their time among these dimensions also seems to be important. The next step is to model wellness at the senior management—or senior resident—level to set an example. Finally, opportunities must be created and supported for individuals to influence and improve their personal wellness.

## Wellness During COVID-19

As the medical world tackled its ongoing battle with physician wellness and prevention of burnout, a new pandemic arrived in 2020: COVID-19. With risk factors to physician burnout being well documented and studies illustrating that greater than 50% of residents either in orthopaedics or another specialty meet the criteria for burnout, the onset of a worldwide call to treat a novel pathogen tipped an already delicate balance into a spiral.^5-7,17^ Studies such as the one undertaken by Kannampallil et al^37^ have demonstrated the deleterious effects that exposure to patients with COVID-19 can have on physician trainees' rates of depression, anxiety, stress, burnout, and professional fulfillment. Further exacerbating the situation, the postponement of elective surgical procedures and in-person conferences, as well as the inability to socialize at work and outside the hospital, during the COVID-19 pandemic changed the landscape of what residency programs had access to regarding wellness initiatives.^38^ Despite the reports of exceedingly high costs attributable to physician burnout in the United States, with estimates between $2.6 and $6.3 billion dollars annually, hospital-sponsored residency events were put on hold as ventilators, PPE, and screening tests became the focus of institutional resources.^39^ Nolte et al^14^ demonstrated the increased emotional burden faced by residents because of the COVID-19 pandemic, with 44.5% of residents reporting that time away from coresidents had a negative effect on overall resident cohesiveness, with junior residents more likely to experience a lack of program cohesion. As junior residents have been shown previously to be at a higher risk of burnout at baseline, this becomes a worrying predicament. In addition, only 41.6% of residents reported nonacademic events held to maintain interaction during the pandemic, whereas 64.2% endorsed weekly communication with their program directors.^14^

Although the harmful effects of the COVID-19 pandemic have been felt throughout all aspects of healthcare and all medical professionals, innovations and adjustments in residency practices because of the COVID-19 pandemic do offer a silver lining regarding wellness. The widespread adoption of online video format for meetings offers the opportunity for easier access to resident lead happy hours, visits with mental health professionals, check-ins with program directors and faculty mentors, and the ability to network with attendings at other programs. Moreover, the ability to attend meetings virtually allows the opportunity to increase time and engagement spent with family and loved ones. As in-person conferences and meetings inevitably return, residents and attendings should continue to emphasize the value of family and personal relationship quality time. Further studies are required to fully understand the potential impact of online video format for meetings and conferences. Given the mass adoption and potential benefits, a hybrid model that allows for both in-person and virtual attendance may offer a balance for those seeking face-to-face interactions with those who would like additional flexibility in their schedules. In addition, the onset of the pandemic may have further increased the emphasis some programs place on resident well-being and the need to promote a culture of wellness and personal resilience.^40^ As we progress in our ability to treat and hopefully prevent the COVID-19 virus, we should take what we have learned with innovations in wellness during the pandemic and combine it with our previous methods of educating, treating, and preventing physician burnout.

## Conclusion

Physician well-being is an important topic that can shape the healthcare landscape over the years to come. Although researchers are beginning to identify the causes, risk factors, and potential interventions for promoting wellness and preventing burnout among orthopaedic surgery residents, there remains a tremendous amount to learn. By incorporating successful strategies from different fields of medicine, nonmedical professions, and learning from our experiences both before and during the COVID-19 pandemic, we can hope to create a more universal wellness curriculum that focuses on a transition from identification of the symptoms to education, treatment, and, eventually, prevention of physician burnout. To accomplish this, specific focus needs to placed on both the individual resident and the system in which they practice, direct person-to-person communication, promoting resident camaraderie, investment from program and departmental leadership, and a culture of inclusiveness, understanding, and acceptance.
